# Multi-target machine learning for predicting mechanical properties of FDM-printed polymer components

**DOI:** 10.1038/s41598-026-49134-6

**Published:** 2026-04-22

**Authors:** A. Lakshumu Naidu, Lakshmana Rao Kalabarige, D. Sandhya Saraswathi, Abhijit Bhattacharya, Pankaj Kumar, Pawan Kumar Singotia, Shyam Sunder Sharma

**Affiliations:** 1 Department of Mechanical Engineering, GMRIT Deemed to be University, Rajam, Andhra Pradesh 532127 India; 2 Department of Computer Science Engineering, GMRIT Deemed to be University, Rajam, Andhra Pradesh 532127 India; 3 Department of Mathematics, Aditya University, Surampalem, Andhra Pradesh 533437 India; 4 Department of Computational Sciences, Brainware University, Kolkata, West Bengal 700125 India; 5 Department of Mechanical Engineering, Raghu Engineering College, Visakhapatnam, Andhra Pradesh 531162 India; 6 Department of Mechanical Engineering, Manipal University Jaipur, Jaipur, Rajasthan 303007 India

**Keywords:** Additive, Polymers, Regression, Prediction, Optimization, Experiments, Manufacturing, Intelligence, Engineering, Materials science, Mathematics and computing

## Abstract

Additive manufacturing using Fused Deposition Modelling (FDM) is increasingly adopted for producing polymer components; however, achieving consistent and repeatable mechanical performance remains a challenge due to process-induced variability and material behavior. Existing studies have predominantly focused on predicting individual mechanical properties using single-output machine learning (ML) models, which limits their ability to capture interdependencies among multiple responses. The present study addresses this gap by investigating whether a Multi-Target Machine Learning (MTML) framework can effectively predict multiple mechanical properties of FDM-fabricated polymer components simultaneously. Experimental datasets were generated from tensile, hardness, and impact tests conducted on specimens fabricated using Polylactic Acid (PLA), Acrylonitrile Butadiene Styrene (ABS), and Polyethylene Terephthalate Glycol-modified (PETG) materials. Several regression-based ML models, including K-Nearest Neighbour (KNN), Decision Tree (DT), Random Forest (RF), Extra Trees (ET), Gradient Boosting (GB), and Extreme Gradient Boosting (EGB), were implemented within both single-target and multi-target learning paradigms. The predictive performance of the models was evaluated using standard statistical metrics, including Mean Absolute Error (MAE), Mean Squared Error (MSE), Root Mean Squared Error (RMSE), and R^2^-score. The results demonstrate strong agreement between predicted and experimental values, with R^2^ values ranging from 0.74 to 0.998, indicating the effectiveness of the MTML framework in capturing complex, non-linear relationships among mechanical responses. The study confirms that the proposed MTML approach improves predictive reliability and modeling efficiency compared to conventional single-output strategies. The findings contribute to advancing data-driven predictive modeling in FDM-based additive manufacturing and provide a robust foundation for future applications in process optimization, quality assessment, and intelligent manufacturing systems. The results demonstrate strong predictive capability, with R^2^ values ranging from approximately 0.74 to 0.998, depending on the material, mechanical property, and regression model. Among the evaluated algorithms, Gradient Boosting Regression (GBR) consistently achieved the highest accuracy for ductility-related properties such as reduction in area and elongation, while Extra Trees Regression (ETR) and GBR showed robust performance for strength-related properties across multiple materials. Overall, the study confirms that the proposed MTML framework improves prediction reliability and modelling efficiency compared to conventional single-output approaches. The findings provide a data-driven foundation for mechanical property estimation in FDM and highlight GBR as the most efficient and reliable regression algorithm within the investigated experimental scope.

## Introduction

Additive Manufacturing (AM), commonly known as 3D printing, is an innovative manufacturing method that produces components layer by layer based on a computer-aided design (CAD) model^1^. This layer-by-layer fabrication technique allows for the creation of intricate geometries, internal features, and custom designs that are challenging to achieve with traditional manufacturing methods. Consequently, AM has become a transformative technology across various engineering and industrial sectors^2^.

In contrast to traditional subtractive manufacturing processes, which involve the progressive removal of material through machining, drilling, or cutting techniques, AM adopts a material-efficient approach by depositing material precisely where it is needed^3^. This methodology not only significantly reduces material wastage but also diminishes the necessity for extensive post-processing, thereby promoting more sustainable and cost-effective production practices^4^.

One of the most notable advantages of additive manufacturing is its capacity for rapid prototyping. Engineers and designers are able to swiftly convert digital models into physical prototypes, enabling them to assess functionality and iteratively refine designs within a condensed timeframe. This accelerated feedback loop significantly reduces product development cycles and fosters increased innovation^5^.

Additive manufacturing has progressed beyond just prototyping and is now a practical option for producing functional and end-use components. Its capability to facilitate low-volume production with high complexity and customization makes it especially appealing to industries such as aerospace, automotive, biomedical engineering, and consumer products. Additive manufacturing encompasses a wide variety of materials, including polymers, metals, ceramics, and composite systems^6^. To effectively process these materials, several techniques have been developed, such as powder bed fusion, directed energy deposition, material extrusion, binder jetting, and vat photopolymerization. Each of these methods comes with its own set of advantages, tailored to the unique properties of the materials and the specific requirements of different applications^7^.

FDM has become widely adopted due to its simplicity, low cost of equipment, and ease of handling materials. FDM operates by extruding thermoplastic filaments through a heated nozzle, depositing material layer by layer to build three-dimensional objects in accordance with the CAD model^8^. The thermoplastic filaments that are commonly utilized in FDM include PLA, ABS, and PETG. These materials are esteemed for their favorable mechanical properties, availability, and compatibility with both desktop and industrial-scale FDM systems^9^.

PLA is often chosen for its biodegradability and ease of printing, while ABS is favored for its enhanced toughness and thermal resistance. PETG combines the benefits of both materials by offering good strength, flexibility, and chemical resistance, making it suitable for a wide range of functional applications^10^. FDM based additive manufacturing significantly contributes to sustainability by minimizing material waste and facilitating decentralized production in proximity to the point of use^11^. This localized manufacturing capability not only reduces transportation requirements but also lowers energy consumption and mitigates associated carbon emissions. Consequently, it fosters environmentally responsible manufacturing practices^12^.

Despite the widespread implementation of FDM in polymer-based additive manufacturing, achieving uniform mechanical performance continues to present significant challenges^13^. These challenges arise from the intricate interactions among material properties, variability induced by the manufacturing process, and the geometry of the specimens. ML methodologies have increasingly been utilized to predict the individual mechanical properties of components fabricated through fused deposition FDM. However, the majority of existing research primarily focuses on single-output models or the independent prediction of mechanical responses. Such methodologies do not adequately account for the interdependence among various mechanical properties that stem from shared microstructural characteristics, including the quality of interlayer bonding, the distribution of porosity, and the thermal history^14^.

To address these limitations, the present study introduces a MTML framework that enables the simultaneous prediction of multiple correlated mechanical properties derived from tensile, hardness, and impact tests. This research presents a systematic evaluation of Multi-Task Multi-Label Models (MTMLM) across three widely utilized thermoplastic materials PLA, ABS, and PETG. This study distinguishes itself from previous work by moving beyond isolated materials or individual responses, utilizing experimentally validated datasets to enhance the reliability of its findings^15^. The innovation of this study is characterized by its physically motivated categorization of correlated tensile responses, a thorough comparison of single-target and multi-target learning methodologies, and a focus on the reliability and generalizability of predictions over mere peak performance. Collectively, these contributions enhance data-driven predictive modeling within the framework of FDM and establish a solid foundation for future applications in intelligent manufacturing^16^.

Ensemble learning techniques enhance prediction accuracy by integrating multiple models, allowing for the capture of complex interactions between process parameters and output responses^17^. Studies related to FDM have demonstrated that these methods outperform traditional regression techniques. Artificial Neural Networks (ANN) have been extensively employed to predict tensile strength, flexural strength, Young’s modulus, and strain behavior of FDM-printed PLA and ABS specimens. Their ability to model highly non-linear systems makes them particularly effective for additive manufacturing applications^18^.

Fuzzy Logic (FL) and Adaptive Neuro-Fuzzy Inference Systems (ANFIS) have also been applied to analyze the influence of critical process parameters such as layer height, infill direction, and print speed on the mechanical behavior of fiber-reinforced and unreinforced thermoplastic materials^19^. Overall, the integration of intelligent, data-driven modeling frameworks with FDM-based additive manufacturing represents a promising pathway toward improving process reliability, enhancing repeatability, reducing manufacturing failures, and optimizing the mechanical performance of printed components. These advancements are essential for expanding the industrial adoption of FDM and realizing its full potential in advanced manufacturing applications^20^.

Furthermore, the growing availability of experimental data from FDM processes has significantly facilitated the adoption of ML techniques in additive manufacturing research^21^. Large datasets generated from tensile, hardness, impact, and flexural tests provide valuable information for training predictive models, enabling more accurate estimation of mechanical properties under varying process conditions. Another important advantage of ML-based approaches is their ability to support multi-objective optimization in FDM. Unlike traditional single-response optimization methods, ML models can simultaneously consider multiple output responses-such as strength, ductility, hardness, and impact resistance—thereby assisting researchers and manufacturers in identifying optimal parameter combinations that balance competing performance requirements^22^.

Recent advances in multi-target and multi-output ML frameworks have further enhanced the capability of predictive modeling in additive manufacturing^23^. These approaches allow the simultaneous prediction of several mechanical properties from a common set of input parameters, improving computational efficiency and capturing interdependencies between different material responses^24^. In addition to mechanical performance prediction, ML models have also been employed for process monitoring, fault detection, and quality assurance in FDM systems^25^. By analyzing printing data and process signals, these models can help identify anomalies, predict potential failures, and support real-time decision-making, thereby improving process robustness and reducing material and time losses^26^.

Overall, the continued integration of ML with FDM-based additive manufacturing is expected to play a critical role in advancing smart manufacturing systems^27^. By enabling predictive analytics, adaptive process control, and data-driven optimization, ML-assisted FDM processes can significantly enhance manufacturing efficiency, product reliability, and scalability, supporting the broader transition toward Industry 4.0-enabled additive manufacturing ecosystems^28^.

From the literature, it has been observed that FDM struggles to achieve consistent and repeatable printing quality in the final product due to variations in process parameters and material properties^29^. Previous studies have typically used multilinear regression models to handle multiple input process parameters of FDM to predict the outcome of only a single mechanical property. However, tensile tests on materials like PLA, ABS, and PETG involve multiple input process parameters and result in multiple output properties, making traditional single-output approaches less effective^30^.

Material extrusion-based additive manufacturing, commonly referred to as FDM, is one of the most widely used additive manufacturing techniques owing to its low equipment cost, minimal material waste, and flexibility in fabricating complex geometries from thermoplastic filaments^31^. However, the layer-by-layer fabrication inherent to material extrusion introduces several performance-related challenges that directly impact component quality. For instance, inadequate interlayer bonding remains a fundamental limitation, often leading to anisotropic mechanical behaviour and reduced structural strength in printed parts (as discussed in recent studies addressing bonding challenges in material extrusion processes). Such bonding issues contribute to porosity and void formation within the printed matrix, which in turn can degrade surface integrity and mechanical characteristics such as hardness and compressive strength^32^. Furthermore, surface roughness arising from the staircase effect and process dynamics frequently necessitates post-processing to meet functional tolerances, which adds cost and complexity to production workflows^33^. Collectively, these performance limitations highlight the complexity of mechanical responses in FDM-fabricated components and underscore the need for data-driven predictive models capable of capturing the influence of multiple interdependent factors.

To address these limitations, there is a need for ML models that can estimate or capture the relationships between multiple input process parameters and multiple target properties simultaneously. Despite the growing application of ML techniques in FDM-based additive manufacturing, several limitations remain in existing studies. Most reported approaches primarily focus on the prediction of single mechanical properties, such as tensile strength or surface quality, often using independently trained models. Such single-output strategies fail to capture the interdependencies among multiple mechanical responses that arise from shared material behavior, process variability, and specimen geometry. Moreover, comparative investigations across multiple commonly used FDM polymers using a unified modeling framework remain limited, restricting the generalizability and practical relevance of current predictive approaches.

In this connection, to address these gaps, the proposed work focuses on developing and applying both single-target and multi-target ML regression models to simultaneously predict key mechanical properties, facilitating more comprehensive quality estimation and process assessment in material extrusion applications^34^. Single-target regression models will be used to analyse experimental data from impact and hardness tests of PLA, ABS, and PETG to understand the relationship between multiple input process parameters and a single mechanical property. Furthermore, propose and evaluate a MTML framework for simultaneously predicting multiple mechanical properties of FDM-fabricated components on tensile test data of the same materials to capture the complex relationships between multiple input process parameters and multiple mechanical properties. The specific objectives of this research are: (i) to construct experimentally validated datasets derived from tensile, hardness, and impact tests of PLA, ABS, and PETG specimens; (ii) to implement and compare multiple single-target and multi-target regression models; and (iii) to assess the capability of MTML models to capture correlated mechanical responses with improved predictive reliability.

The novelty of this work lies in the systematic application of MTML to jointly predict correlated tensile properties, alongside hardness and impact behavior, across multiple polymer materials within a consistent experimental and modeling framework. By moving beyond isolated property prediction, this study provides deeper insight into the coupled mechanical behavior of FDM-printed components and demonstrates the potential of MTML as a robust data-driven tool for performance estimation and manufacturing decision support.

## Experimental setup

The AM process involves a series of well-defined steps, beginning with the creation of a 3D model using Computer-Aided Design (CAD) software^35^. After finalizing the design, the 3D model is exported in STL (stereolithography) format. The STL file is then imported into slicing software like Cura, where various analyses are conducted, such as support structure generation, profile cutting, and defining print settings. These settings are then converted into G-CODE, a machine-readable format, which is transferred to the 3D printer for fabrication^36^.

This experimental study focused on the FDM^37^ process using PLA, ABS, and PETG fiber-reinforced composite filaments. The aim is to thoroughly explore the complexities of 3D printing with these advanced composite materials. The process flow of FDM within AM^38,39^, as illustrated, outlines the steps involved in producing 3D parts using PLA and ABS fiber-reinforced filaments.

The primary goal of this research is to identify the optimal process parameters for FDM^40^ in order to maximize the mechanical properties-such as tensile strength, impact strength, and hardness of PLA and ABS Fiber-reinforced composites to achieve maximum precision and control. This work stands out for its creative combination with ML methods. By leveraging advanced data-driven algorithms^41^, this work seeks to develop complex relationships between various FDM printing parameters and the resulting mechanical characteristics of the printed components.

This fusion of modern materials, cutting-edge manufacturing processes, and artificial intelligence (AI) represents a transformative leap in the field of FDM, driving significant advancements under the broader umbrella of AM^42^.

**Fig. 1:**
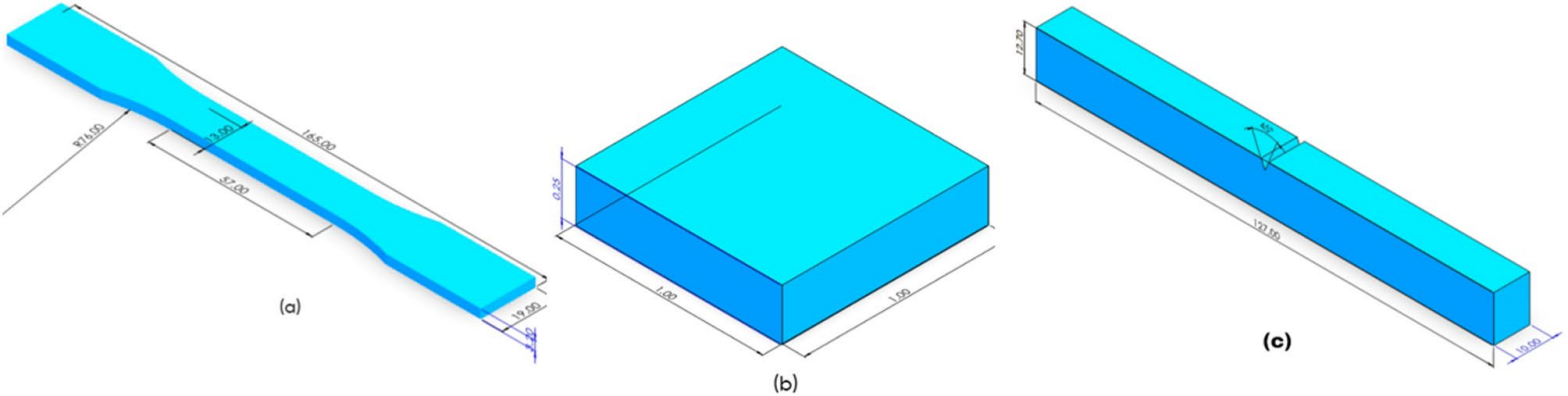
3D CAD Model of the (**a**) Tensile Specimen, (**b**) Hardness Specimen and (**c**) Impact Specimen.

The implications of this research work extended far beyond the scope of this experimentation. The application of PLA, ABS and PETG thermoplastic composites, guided by ML algorithms, opens new possibilities for enhancing the mechanical properties of 3D-printed parts. The results of this work have the potential to redefine how we conceptualize, design, and manufacture functional objects across a range of industries, including aerospace engineering, automotive manufacturing, medical devices, and customized consumer products^43^. This synergy between materials science, advanced manufacturing, and ML is poised to revolutionize the future of AM and FDM, shaping the development of high-performance components with unprecedented precision and efficiency. The items shown in Fig. 1 are the specimens or 3D CAD models^44^ used in this work. The tensile specimen as shown in Fig. 1a, specimen for hardness shown in Fig. 1b, and specimen for impact test in Fig. 1c.

**Fig. 2:**
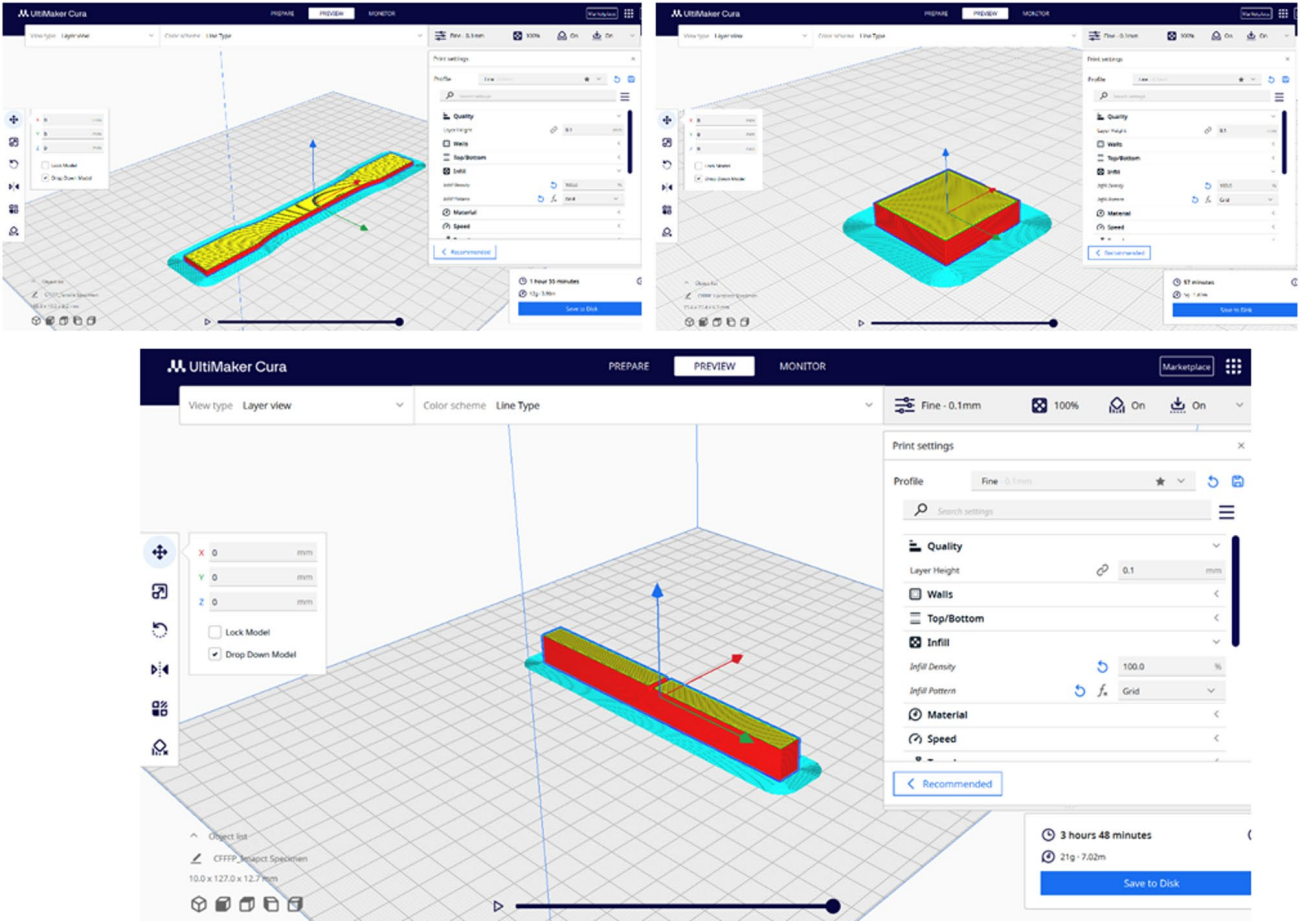
Slicing analysis in Cura software.

Furthermore, The CAD models are loaded into Cura Software, and the functionality of the program with regard to slicing analysis, support generation, and toolpath optimization is thoroughly examined as reported in Fig. 2. The infill percentage^45^, infill pattern^46^, and G-CODE^47^ generation are important phases during the slicing process. The infill percentage from 0 to 20% is low, 20% to 50% is medium, and 50% to 100% is high. This work considered infill percentage from 5 to 100%.

**Fig. 3:**
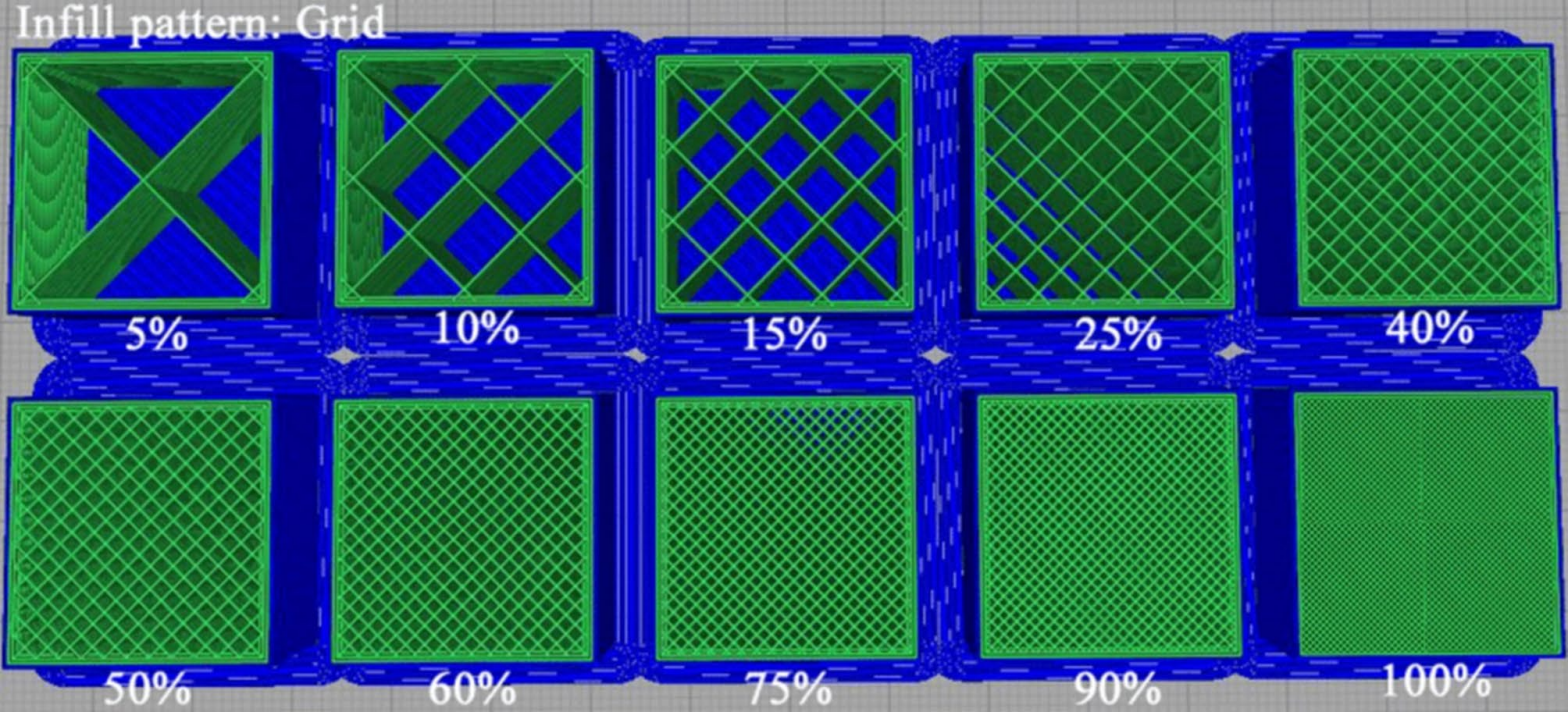
Infill pattern of the specimen with different percentages.

Furthermore, this work uses the grid or honeycomb infill pattern with filling percentages from 5 to 100% as reported in Fig. 3. Moreover, Table 1 presents the generated G-CODE for this work.

**Table 1:** Generated G-code for the fabrication process in this study.

Starting code	Ending code
**Flavor:** Marlin	TIME ELAPSED: 4037.977846
**Total Printing Time:** 4037 s	G1 F1500 E3618.79688
**Filament Used:** 11.9433 m	M140 S0
**Layer Height:** 0.4 mm	M107
**Dimensions:**	M104 S0
Minimum X Coordinate: 71.8 mm	M140 S0
Minimum Y Coordinate: 113.72 mm	Retract the filament
Minimum Z Coordinate: 0.3 mm	G92 E1
Maximum X Coordinate: 278.2 mm	G1 E-1 F300
Maximum Y Coordinate: 140.12 mm	G28 X0 Y0
Maximum Z Coordinate: 9.9 mm	M84
**Slicing Software:** Generated with Cura Steam Engine version 4.11.0	M82 absolute extrusion mode
This information can be useful for understanding the parameters and capabilities of the print	M104 S0
	End of G-code

### Printing and testing of specimens

**Fig. 4:**
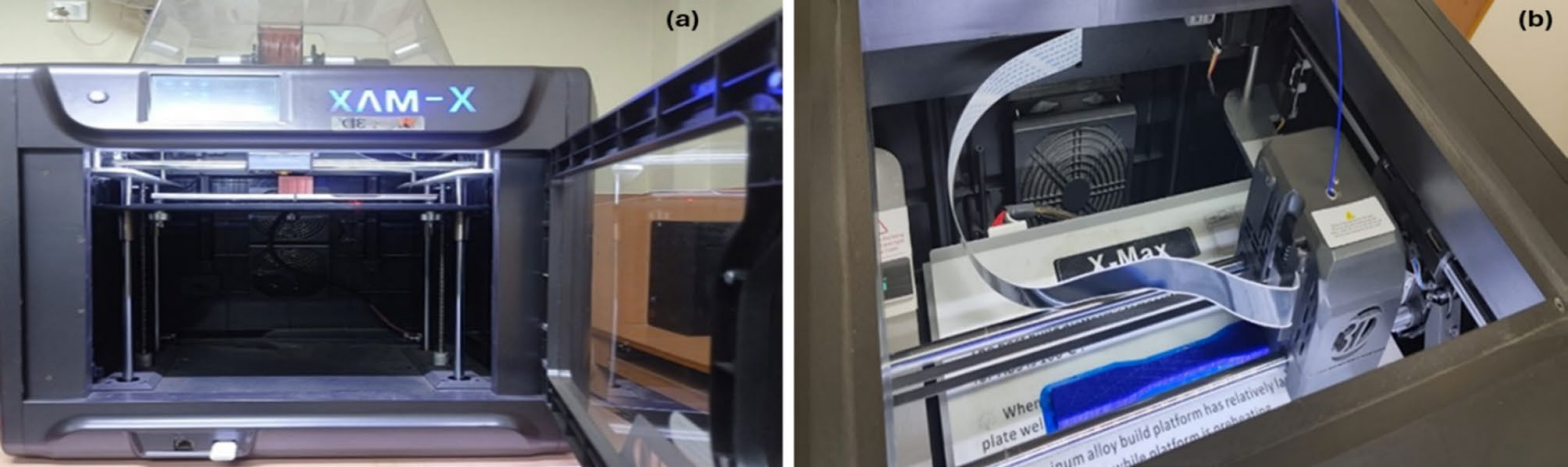
(**a**) 3D Printer and (**b**) Printed specimen.

Figure 4a shows the 3D printer^48^ used in this work for printing specimens as reported in Fig. 4b. The G-Code generated during the slicing process fed to the 3D printer to make the digital models as physical objects as reported in Fig. 4b. The printed specimen underwent three tests such as Tensile, Hardness, and Impact tests using instruments. The Universal Testing Machine (UTM)^49^ for tensile test to determine the maximum stress that a material withstands. This test is useful to understand the behaviour of a material under load. Similarly, the Rockwell Hardness Test Machine^50^ is reported used to determine the printed material resistance to deformation, indentation, or scratching. The hardness test of a material helps in selecting suitable materials for specific applications. Furthermore, the Impact test machine^51^ reported measures material’s ability to absorb energy and tests fracture resistance capacity when subjected to a sudden load or impact.

## ML optimization

ML constitutes a subset of artificial intelligence (AI) that concentrates on the creation of algorithms and models that empower computers to acquire knowledge and make predictions or decisions without the need for explicit programming. Essentially, it represents the science of enabling computers to learn from data and enhance their performance on specific tasks over time^52^.

ML algorithms are mathematical models and techniques used to analyse and learn from data. These algorithms can be categorized into supervised learning, unsupervised learning, and reinforcement learning, among others^53^.

ML techniques are being increasingly utilized in research related to additive manufacturing to effectively model the intricate, non-linear relationships among material properties, variability induced by the fabrication process, and mechanical performance^54^. Within the framework of FDM, the mechanical properties of printed polymer components-such as tensile strength, elongation, hardness, and impact resistance-are influenced by a myriad of factors. These include geometric attributes, material characteristics, and process-induced effects, such as interlayer bonding and porosity. The complex interactions among these variables pose significant challenges for capture using traditional analytical or statistical models^55^.

In this study, the application of ML optimization is directed specifically towards datasets generated experimentally from tensile, hardness, and impact testing of FDM, 3D printed specimens composed of PLA, ABS, and PETG. The primary objective is to assess the efficacy of both single-target and multi-target regression models in accurately predicting mechanical responses based on the input features observed in the experimental data^56^. The proposed framework utilizes ensemble and tree-based regression algorithms to effectively capture the interdependent effects of multiple variables on mechanical performance. This approach facilitates a more accurate, data-driven estimation of the behavior of FDM components. The application of ML within this specific context serves as the foundation for the subsequent development, training, and comparative evaluation of the model presented in this section^57^.

All specimens used in this study were fabricated using a material extrusion-based FDM 3D printer. The printer operates with thermoplastic filament feedstock and is equipped with a temperature-controlled extrusion system suitable for printing PLA, ABS, and PETG materials. Specimens were prepared using commercially available neat thermoplastic filaments, and all prints were generated from CAD models sliced using Cura software^58^.

A consistent set of printing parameters was employed to ensure repeatability across materials, while selected parameters were varied as part of the experimental dataset. Layer height, nozzle diameter, raster pattern, build orientation, and printing speed were maintained constant for all specimens to minimize variability arising from machine settings. The infill percentage was varied systematically over a defined range and recorded as an input feature, as it directly influences mechanical performance^59^. The ranges of printing parameters used in the study are summarized in the corresponding table.

Mechanical testing was conducted in accordance with established international standards. Tensile tests were performed following ASTM D638, hardness measurements were conducted in accordance with ASTM E384, and impact testing was carried out following ASTM D256. Specimen geometries were designed to comply with the dimensional requirements specified in these standards to ensure consistency and comparability of results^60^.

In this study, the machine learning models utilize experimentally observed variables as input features, including geometric measurements and post-fabrication specimen characteristics obtained during standardized testing procedures^61^. These variables were intentionally selected because they capture the combined influence of material behaviour, printing conditions, and structural characteristics of the fabricated specimens^62^. Since mechanical responses such as tensile strength, elongation, hardness, and impact resistance depend strongly on specimen geometry and post-processing deformation behaviour, incorporating these experimentally measured attributes enables the models to better capture the complex nonlinear relationships between structural characteristics and mechanical performance. Therefore, the proposed modelling framework is primarily intended for post-fabrication performance estimation and data-driven analysis of mechanical behaviour, rather than real-time prediction during the printing process.

### Preparation of the dataset

The results of experimental tests such as tensile, hardness, and impact obtained through different properties reported in Table 2 of 3D printing of test specimens (reported in Figs. 2 and 3) of Natural Fiber reinforced materials such as PLA, ABS, and PETG were collected as nine datasets *d*_1_ to *d*_9_ respectively^63^. The experimental results of PLA material were indicated as $d_{1}$ with 92 instances for Tensile, $d_{2}$ with 38 instances for Impact, and $d_{3}$ with 48 instances for Hardness respectively. Similarly, experimental results of ABS for Tensile, Impact, and Hardness were indicated as $d_{4}$ with 91 instances, $d_{5}$ with 41 instances, and $d_{6}$ with 49 instances, respectively. Moreover, experimental results for Tensile, Impact, and Hardness of PETG were indicated as $d_{7}$ with 92 instances, $d_{8}$ with 49 instances, and $d_{9}$ with 50 instances, respectively.

**Table 2:** The parameters/properties/features of Tensile, Impact, and Hardness tests.

(a) Properties of tensile test	(b) Properties of hardness test	(c) Properties of impact test
Independent input properties of Tensile	Independent input properties of Hardness	Independent input properties of Impact
1. Specimen width (mm) 2. Specimen thickness (mm) 3. Initial gauge length (mm) 4. Preload (KN) 5. Max load (KN) 6. Max elongation (mm) 7. Cross-sectional area (mm) 8. Final width (mm) 9. Final thickness (mm) 10. Final gauge length (mm) 11. Final area (mm^2^)	1. Position (H1) 2. Position (H2) 3. Position (H3) 4. Position (H4) 5. Position (H5) 6. Infill percentage	1. Infill percentage 2. Cross-section area (mm^2^) 3. Energy Units (J)

Dependent output properties tensile	Dependent output properties hardness	Dependent output properties impact
1. Load at peak (KN) 2. Tensile strength (N/mm^2^) 3. % reduction area (%) 4. % elongation (%)	1. Vickers hardness number	1. Impact strength (J/A) N/MM
Instances		
94	49	41

The tensile test includes 11 input properties and 4 output properties, as shown in Table 2, and consists of 94 instances. The hardness test has 6 input features and 1 output feature, as illustrated in Table 2, comprising 49 instances. Lastly, the impact test features 3 input parameters and 1 output parameter, also detailed in Table 2, with a total of 41 instances. The statistics of the datasets $d_{1} ,d_{4} ,\;{\mathrm{and}}\;d_{7}$ indicate experimental results of tensile tests of PLA, ABS, and PETG materials as reported in Table 3, and statistical description of datasets $d_{3} ,d_{6} ,\;{\mathrm{and}}\;d_{9}$ indicates results of hardness tests of PLA, ABS, and PETG materials as reported in Table 4, thereafter Table 5 reported statistics of the datasets $d_{2} ,d_{5} ,\;{\mathrm{and}}\;d_{8}$ represent experimental results of impact test of PLA, ABS, and PETG materials respectively.

**Table 3:** Tensile test experimental data of PLA, ABS, and PETG.

		Final width (mm)	Final thickness (mm)	Final gauge length (mm)	Final area (mm)	Load at peak (kN)	Tensile strength (N/mm^2^)	% reduction area (%)	% elongation (%)
PLA-tensile ($d_{1}$)	Count	92.00	92.00	92.00	92.00	92.00	92.00	92.00	92.00
	Mean	11.51	9.47	62.00	109.03	4.05	33.76	9.14	3.34
	Std	0.26	0.29	0.65	4.12	0.52	4.35	3.44	1.08
	Min	11.10	9.00	60.90	99.90	3.01	25.10	1.83	1.50
	25%	11.30	9.20	61.50	106.16	3.65	30.40	6.73	2.50
	50%	11.50	9.50	61.95	109.15	3.99	33.25	9.05	3.25
	75%	11.70	9.70	62.60	111.93	4.53	37.77	11.54	4.33
	Max	11.90	9.90	63.00	117.81	4.96	41.33	16.75	5.00
ABS-tensile ($d_{4}$)	Count	91.00	91.00	91.00	91.00	91.00	91.00	91.00	91.00
	Mean	11.51	9.45	62.01	108.82	3.53	29.45	9.32	3.35
	Std	0.26	0.29	0.65	4.15	0.21	1.77	3.45	1.09
	Min	11.10	9.00	60.90	99.90	3.12	26.00	1.83	1.50
	25%	11.30	9.20	61.55	105.91	3.46	28.83	6.97	2.58
	50%	11.50	9.50	62.00	108.78	3.57	29.75	9.35	3.33
	75%	11.70	9.70	62.60	111.63	3.69	30.71	11.74	4.33
	Max	11.90	9.90	63.00	117.81	3.98	33.17	16.75	5.00
PETG-tensile ($d_{7}$)	Count	92.00	92.00	92.00	92.00	92.00	92.00	92.00	92.00
	Mean	11.48	9.37	62.08	107.56	3.68	30.65	10.37	3.46
	Std	0.30	0.35	0.62	5.25	0.22	1.87	4.38	1.04
	Min	10.60	8.16	60.80	93.84	3.12	26.00	1.83	1.33
	25%	11.30	9.17	61.60	104.15	3.58	29.83	6.58	2.67
	50%	11.50	9.40	61.90	107.90	3.75	31.25	10.09	3.17
	75%	11.70	9.60	62.63	112.10	3.82	31.83	13.21	4.37
	Max	11.90	9.90	63.20	117.81	3.98	33.17	21.80	5.33

**Table 4:** Hardness test experimental data of PLA, ABS, and PETG.

		Position (H1)	Position (H2)	Position (H3)	Position (H4)	Position (H5)	Infill percentage	Vickers hardness number
PLA-hardness ($d_{3}$)	Count	48.00	48.00	48.00	48.00	48.00	48.00	48.00
	Mean	17.56	17.75	17.79	17.92	17.83	78.96	17.77
	Std	1.13	0.85	0.82	0.71	0.64	12.11	0.70
	Min	15.50	15.80	16.10	16.20	16.40	60.00	16.08
	25%	16.75	17.13	17.40	17.30	17.40	70.00	17.38
	50%	17.55	17.85	17.85	17.90	17.85	77.50	17.90
	75%	18.25	18.30	18.33	18.30	18.30	90.00	18.11
	Max	19.80	19.50	19.60	19.50	18.90	100.00	19.18
ABS-hardness ($d_{6}$)	Count	49.00	49.00	49.00	49.00	49.00	49.00	49.00
	Mean	17.70	17.78	17.71	16.71	17.47	81.49	17.47
	Std	1.31	1.35	1.41	1.23	1.49	15.34	1.07
	Min	14.30	14.60	14.20	14.10	13.80	8.00	14.44
	25%	16.90	17.20	16.80	15.90	16.40	75.00	16.98
	50%	17.90	18.00	17.90	16.60	17.50	85.00	17.64
	75%	18.70	18.70	18.90	17.50	18.70	90.00	18.16
	Max	19.70	19.80	19.70	19.80	19.60	100.00	19.00
PETG-hardness ($d_{9}$)	Count	50.00	50.00	50.00	50.00	50.00	50.00	50.00
	Mean	14.84	14.86	14.78	14.83	14.86	79.99	14.83
	Std	0.58	0.58	0.56	0.62	0.55	18.52	0.26
	Min	13.80	13.80	13.80	13.80	13.80	6.50	14.24
	25%	14.30	14.50	14.35	14.30	14.53	75.00	14.65
	50%	14.70	14.70	14.60	14.60	14.70	85.00	14.86
	75%	15.28	15.30	15.20	15.38	15.30	90.00	15.00
	Max	15.90	15.90	15.80	15.90	15.90	100.00	15.46

**Table 5:** Impact test experimental data of PLA, ABS, and PETG.

		Infill percentage	Cross-section area (mm^2^)	Energy (J)	Impact strength (J/A) N/MM
PLA-impact ($d_{2}$)	Count	38.00	38.00	38.00	38.00
	Mean	79.74	100.00	2.03	20.29
	Std	12.78	0.00	0.52	5.25
	Min	60.00	100.00	1.00	10.00
	25%	70.00	100.00	1.60	16.00
	50%	80.00	100.00	2.00	20.00
	75%	90.00	100.00	2.48	24.75
	Max	100.00	100.00	3.00	30.00
ABS-impact ($d_{5}$)	Count	41.00	41.00	41.00	41.00
	Mean	79.39	100.00	1.86	18.59
	Std	13.47	0.00	0.75	7.46
	Min	60.00	100.00	0.50	5.00
	25%	65.00	100.00	1.20	12.00
	50%	80.00	100.00	2.00	20.00
	75%	90.00	100.00	2.40	24.00
	Max	100.00	100.00	3.10	31.00
PETG-impact ($d_{8}$)	Count	49.00	49.00	49.00	49.00
	Mean	81.73	100.00	2.26	22.59
	Std	13.45	0.00	0.40	4.00
	Min	60.00	100.00	1.09	10.93
	25%	70.00	100.00	1.89	18.90
	50%	85.00	100.00	2.36	23.60
	75%	90.00	100.00	2.56	25.60
	Max	100.00	100.00	2.85	28.50

### Single and multi-target regression ML models

**Fig. 5:**
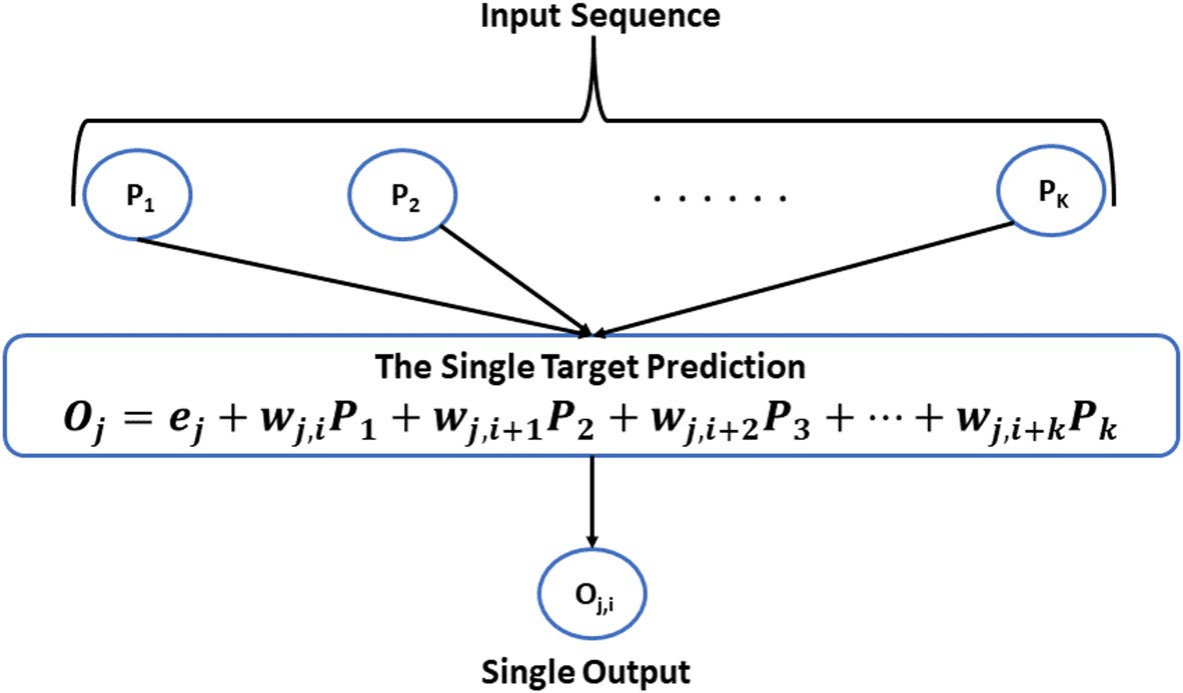
Single target regression model.

As shown in Fig. 5, the supervised learning involves training models to predict the value of a single target attribute $O_{j}$ which can be categorical or numeric, for a given input. The single target model builds a single equation to predict a specified single dependent variable $O_{j}$ where j = i = 1. Then, the $e_{j}$ is an error, $P_{1}$ to $P_{k}$ are input features, and $w_{j,i}$ to $w_{j,i + k}$ are coefficients of *j*th target (dependent) variable. However, there are situations where a single input sample is associated with multiple outputs. In such cases, Multi-target (MT) learning is used, which is a generalization of single-target (ST) learning that involves predicting all the multiple outputs at the same time^64^.

**Fig. 6:**
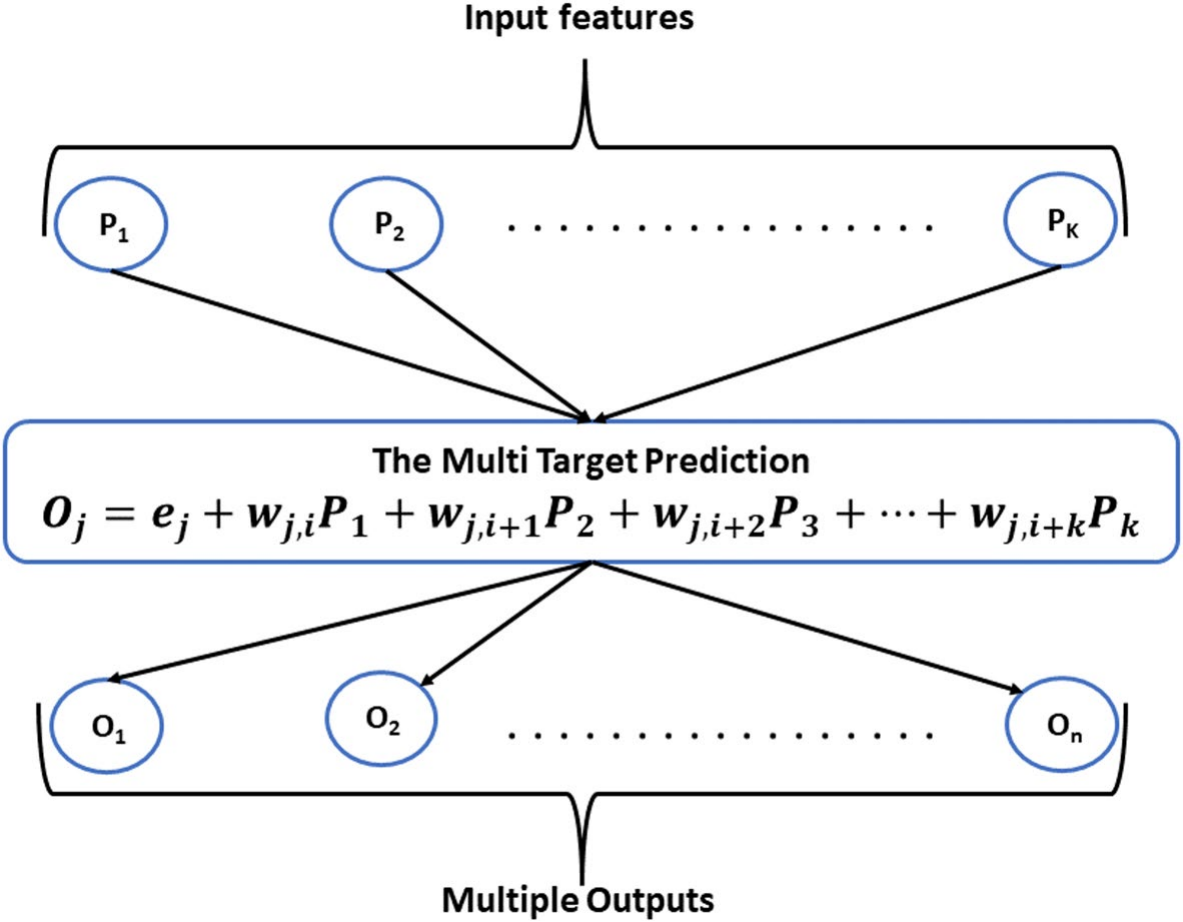
Multi target regression model.

Multi output regression is a technique used to predict multiple outputs that depend on the same inputs as well as on one another. Because these outputs are often interrelated, it is essential to use a model that can predict them simultaneously instead of independently. This approach, known as multitarget output regression, is particularly beneficial when the outputs are interdependent or when predicting them at the same time is more efficient than handling each output separately. Furthermore, multitarget regression can sometimes provide more information about the relationship between the inputs and outputs, which can lead to more accurate predictions. Multi-output regression^65^, also referred to as multi-target, multi-variate, or multi-response regression, is a type of ML where the goal is to predict multiple outputs or target variables simultaneously, as shown in Fig. 6. The multioutput model builds multiple equations to predict multiple dependent variables $O_{j}$ as shown in Fig. 6. Where, $j = 1$ to $n$, $i = 1 to k$, the $O_{j}$ indicates equation for *j*th dependent or target variable, and $w_{j,i}$ is an *i*th coefficient for *j*th target variable^66^.

In this work, the sklearn module of Python is used to implement multi-target regression models. It provides a provision to implement multitarget models in two ways such as using direct multioutput regression models and using a Multi-Output-Regressor model, which makes an ML model a multitarget model. Hence, K-Nearest Neighbor Regression (KNN), Decision Tree Regression (DTR), Random Forest Regression (RFR), and ETR models which support multiple outputs directly, were employed in this work. In addition, GBR and Xgboost Regression (XGB) were made as multitarget regression models using the Multi Output Regressor model.

In this study, DTR is considered as a baseline regression model because of its relatively simple structure and interpretability compared with more advanced ensemble methods. The performance of DTR provides a reference point for evaluating the effectiveness of more sophisticated ensemble learning algorithms such as Random Forest, Extra Trees, Gradient Boosting, and Extreme Gradient Boosting. By comparing these models under the same experimental datasets, the study demonstrates how ensemble-based approaches can better capture complex nonlinear relationships between input parameters and mechanical responses in FDM-fabricated components.

### The single and multi-target ensemble regression model

The proposed approach has six steps scaling, data splitting, training, testing, performance evolution, and comparison analysis (Results and Discussion) as reported in Fig. 6. It employed six ML models such as KNN, DTR, RFR, ETR, GBR, and XGB to evaluate the nine datasets $d_{1} to d_{9}$. These models built $T_{KNN}$, $T_{DTR}$, $T_{RFR}$, $T_{ETR}$, and $T_{XGB}$ respectively after training, and then the predicted output for test data such as $P_{KNN}$, $P_{DTR}$, $P_{RFR}$, $P_{ETR}$, and $P_{XGB}$ of each model was evaluated with performance metrics such MAE, MSE, RMSE, and R^2^-score which was represented as $E_{KNN}$, $E_{DTR}$, $E_{RFR}$, $E_{ETR}$, and $E_{XGB}$ respectively. Each phase of the proposed approach is as follows^67^.

#### Scaling

The datasets under consideration do not contain any null values; however, the input feature values are presented on differing scales. It is important to note that training models with features that exhibit substantial discrepancies in scale may lead to imbalanced contributions during the learning process and model fitting. Consequently, such inconsistencies have the potential to adversely affect the overall performance of the model. Therefore, it is imperative to implement a standardization method, such as Z-score normalization, for the input features, as described in Eq. 1. In this equation, the variable represents the input feature, signifies the mean of the input feature, denotes the number of instances, and indicates the standard deviation.

1 $$Z = \frac{{x_{i} - \mu_{x} }}{\sigma }$$

where $1 \le i \le N$, mean $\mu_{x} = \frac{1}{N}\sum\nolimits_{i = 1}^{N} {x_{i} }$ and standard deviation $\sigma = \sqrt {\frac{1}{N}\sum\nolimits_{i = 1}^{N} {\left( {x_{i} - \mu_{x} } \right)^{2} } }$.

#### Data splitting, model training and testing

**Fig. 7:**
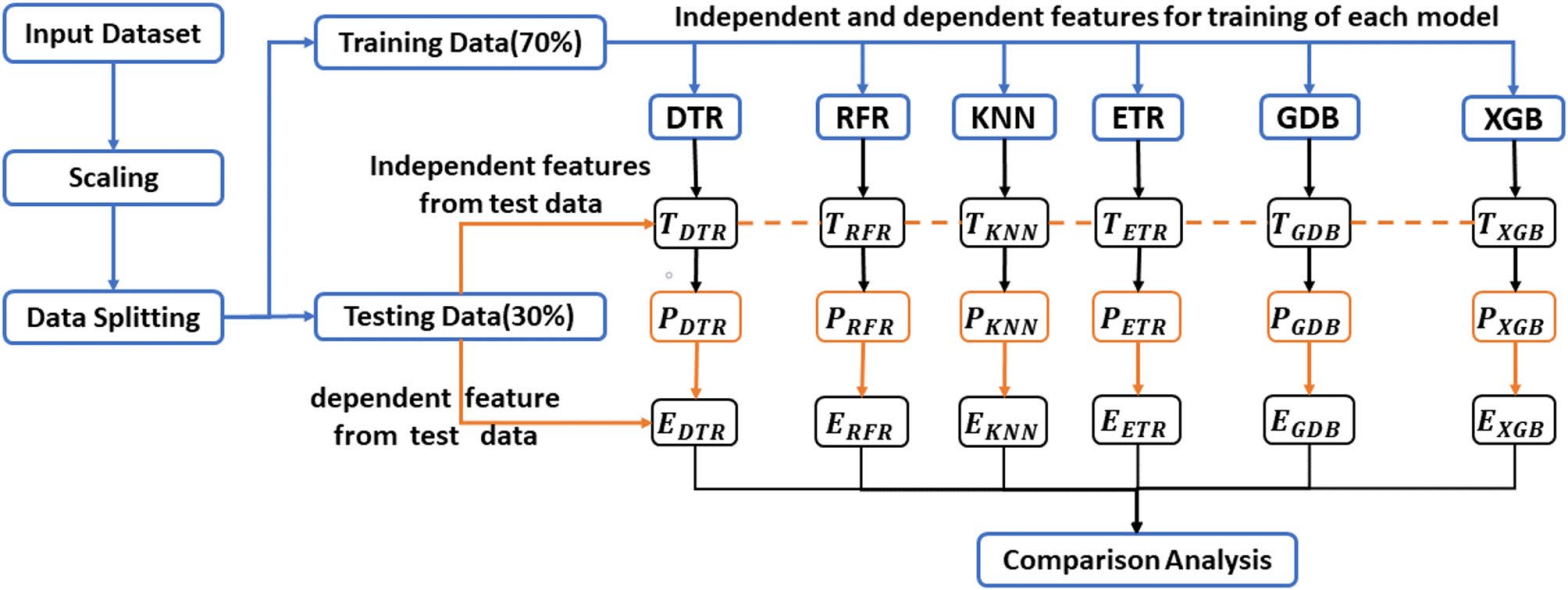
Proposed single and multitarget ensemble regression model.

The scaled data of datasets $d_{1} to d_{9}$ divided into training and testing with 70%:30% ratio. The ML models were trained with 70% of training data and tested with 30% of test data. The trained models such as $T_{DTR} ,T_{RFR} ,T_{KNN} , T_{ETR} , T_{GDB} ,\;{\mathrm{and}}\;T_{XGB}$ were tested with 30% of independent test data and obtained predicted output such as $P_{DTR} ,P_{RFR} ,P_{KNN} , P_{ETR} , P_{GDB} ,\;{\mathrm{and}}\;P_{XGB}$ as shown in Fig. 7. Tensile datasets $d_{1} ,d_{4} ,\;{\mathrm{and}}\;d_{7}$ of three materials has six targets as reported in Table 2 (1st Column) and understood that the dependent features LOAD AT PEAK (Kn) and TENSILE STRENGTH (N/mm^2^) are related with each other. Similarly, % REDUCTION AREA (%) and % ELONGATION (%) are related to each other. Hence, each ML model was trained on each two targets once. In such a way, one model was built, trained, and tested for a single pair of targets. In this way, two different models for a same ML model were built for a tensile test of one material and altogether six different models for a same ML model were created for all three materials. In this way, a total of twelve models were built and analysed. Whereas, each model was trained and tested once on datasets for hardness $d_{3} ,d_{6} ,\;{\mathrm{and}}\;d_{9}$ and impact $d_{2} ,d_{5} ,\;{\mathrm{and}}\;d_{8}$ these datasets have only one dependent feature such as VICKERS HARDNESS NUMBER and Impact strength (J/A) N/MM as shown in Table 2 (2nd and 3rd Column respectively).

#### Model evolution

The prediction performance of ML models was evaluated with 30% of actual test data through MAE, MSE, RMSE, and Coefficient of Determination (R^2^-Score) as described below. Here, the notations shown in Table 6 were followed in the calculation of performance metrics as shown in Fig. 7.

**Table 6:** The Notations used in this work.

Notations	Description
$I_{n}$	The number of instances in dataset
$O^{n}$	The target number
Targets	4 targets in $d_{1} ,d_{4} ,\;{\mathrm{and}}\;d_{7}$ but operated two targets at once from each dataset and n = 1 for remaining datasets
$O_{i}^{j}$	The *i*th actual value of *j*th target
$\hat{O}_{i}^{j}$	The *i*th predicted value of *j*th target
$\underline {O}_{i}^{j}$	Mean of actuals of *j*th target such as $\frac{1}{{I_{n} }}\sum\nolimits_{i = 1}^{{I_{n} }} {O_{i}^{j} }$
$e_{ij}$	Error value measured as $O_{i}^{j} - \hat{O}_{i}^{j}$
$MAE_{j}$	The MAE of *j*th target
$MSE_{j}$	The MSE of *j*th target
$RMSE_{j}$	The RMSE of *j*th target
$R_{j}^{2}$	The Coefficient of Determination of *j*th target

*MAE*: The summation of absolute difference between actual and predicted values average is known as mean absolute error of one target output. Furthermore, the average of all target MAEs shown in the below equation is known as AMSE.

2 $$MAE = \frac{1}{{I_{n} }}\mathop \sum \limits_{i = 1}^{{I_{n} }} \left| {e_{ij} } \right|$$

*MSE*: The MSE is a summation of square of each error value $e_{ij}$ divided with number of samples as shown below. The mean of all three target MSE values is called as AMSE as shown in below equation.

3 $$AMSE = \frac{1}{{O^{n} }}\mathop \sum \limits_{j = 1}^{{yO^{n} }} MSE_{j}$$

where $$MSE_{j} = \frac{1}{{I_{n} }}\sum\nolimits_{i = 1}^{{O_{n} }} {\left( {e_{ij} } \right)^{2} }$$.

*RMSE*: The RMSE measures how best the residuals spread around the actual or line of best fit. It is nothing but square root of MSE and the average all target RMSE values in known as ARMSE as shown in below equation.

4 $$RMSE = \frac{1}{{O^{n} }}\mathop \sum \limits_{j = 1}^{{O^{n} }} \sqrt {MSE_{j} }$$

*Coefficient of determination (Average R*^*2*^*-score)*: The R^2^ score value lies in between 0 to 1. The model performance is said to be good when its value is close to 1. Its value for one target output is calculated as in below equation and the average of all target R^2^ scores is known as average R^2^-Score as shown in below equation. It says how much proportion of variance deviation is observed in describing actual values.

5 $$Average\;R^{2} = \frac{1}{{O^{n} }}\mathop \sum \limits_{j = 1}^{{O^{n} }} R_{j}^{2}$$

where $$R_{j}^{2} = \frac{1}{{O^{n} }}\sum\nolimits_{j = 1}^{{O^{n} }} {1 - \frac{{\mathop \sum \nolimits_{i = 1}^{{I_{n} }} \left( {e_{ij} } \right)^{2} }}{{\mathop \sum \nolimits_{i = 1}^{{O_{n} }} \left( {O_{i}^{j} - \overline{O}_{i}^{j} } \right)^{2} }}}$$.

## Results

**Fig. 8:**
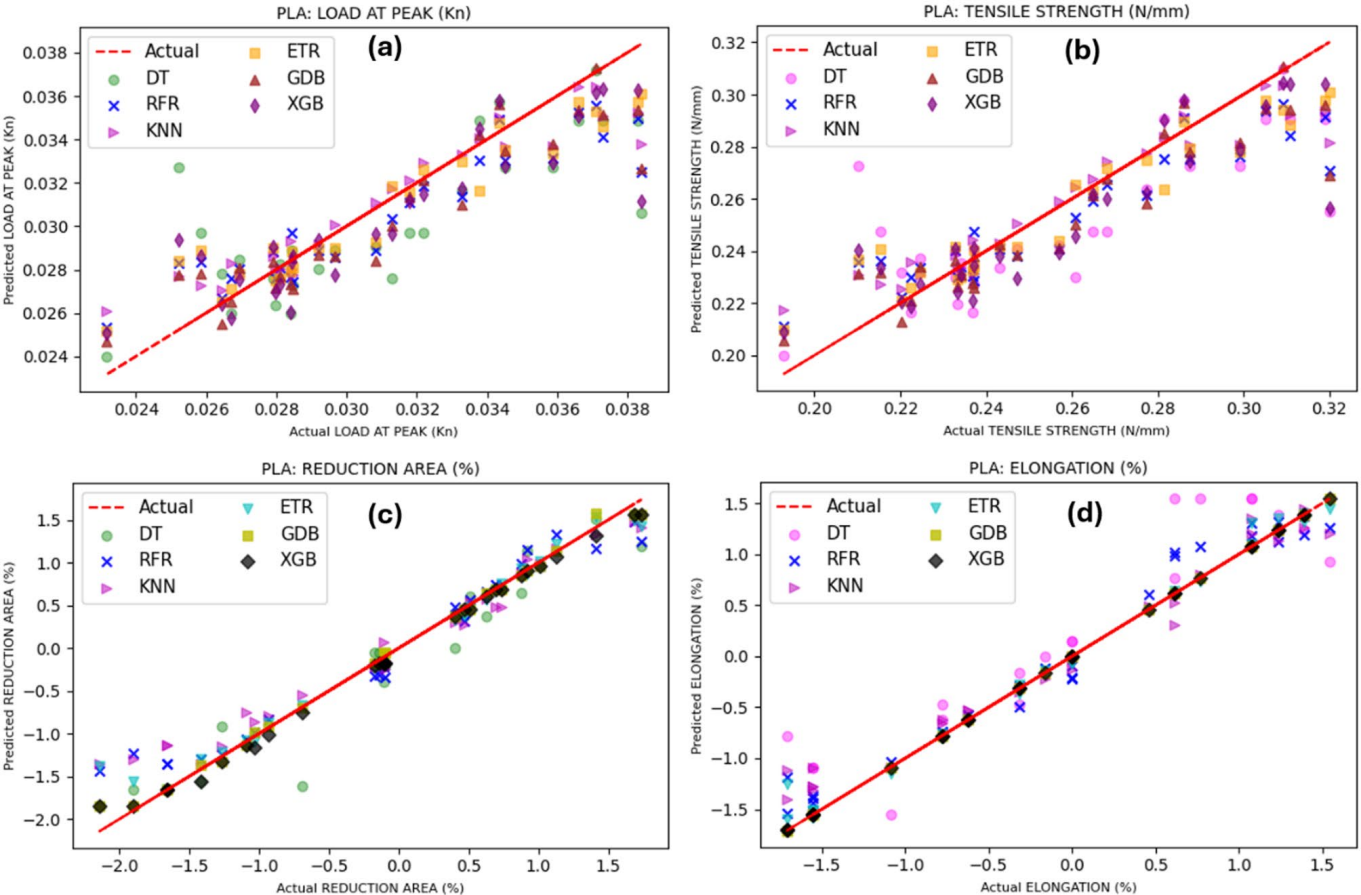
Comparison of actual vs predicted of PLA material: (**a**) Load at Peak, (**b**) Tensile Strength, (**c**) Reduction Area (%), and (**d**) Elongation (%).

**Fig. 9:**
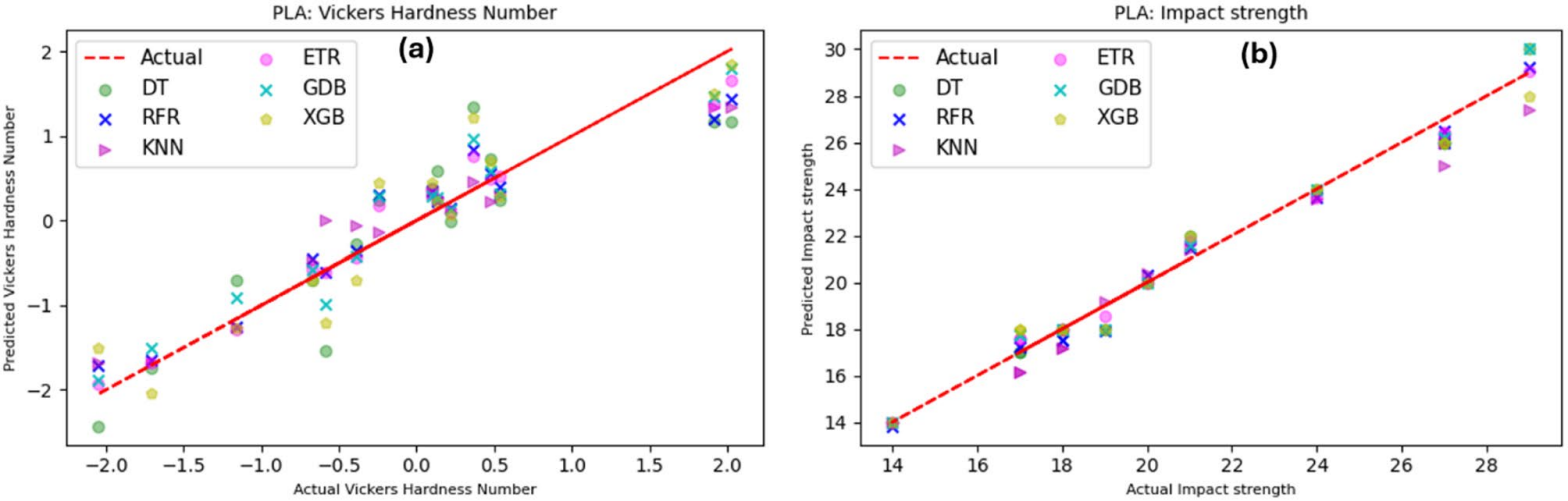
Comparison of actual vs predicted of PLA material: (**a**) Hardness, and (**b**) Impact.

The models such as DT, RF, KNN, ETR, GDB, and XGB were trained with 70% and tested with 30% experimental data (recorded as nine datasets from $d_{1}$ to $d_{9}$) of Tensile, Hardness and Impact tests conducted on three specimens prepared based on three materials PLA, ABS, and PETG as reported in Figs. 8a, b, c, d and  9a, b. The trained models were evaluated through MAE, MSE, RMSE, and R^2^ as reported in Eqs. 1, 2, 3, and 4 respectively. Table 7 (Tensile Test) and Table 8 (Hardnes and Impact Test) presents performance of all ML models on all three specimens. The results of ML models for Tensile, Impact and Hardness tests of three materials PLA, ABS, and PETG were good with less error rate and high R^2^ score.

**Table 7:** Tensile test: load at peak, tensile strength, reduction area (%), and elongation (%).

		PLA				ABS				PETG			
		Performance metrics				Performance metrics				Performance metrics			
Load at peak	Models	MAE	MSE	RMSE	R^2^	MAE	MSE	RMSE	R^2^	MAE	MSE	RMSE	R^2^
	DT	0.001995	0.002724	0.000007	0.590362	0.000801	0.001073	0.000001	0.723862	0.000730	0.000879	0.0000008	0.773773
	RF	0.001468	0.002017	0.000004	0.775444	0.001027	0.001244	0.000002	0.628581	0.000616	0.000840	0.0000007	0.793313
	KNN	0.001066	0.001530	0.000002	**0.870692**	0.000955	0.001233	0.000002	0.635516	0.000785	0.001221	0.0000015	0.563731
	ETR	0.001205	0.001546	0.000002	0.867994	0.000741	0.001038	0.000001	**0.741528**	0.000611	0.000880	0.0000008	0.773463
	GDB	0.001344	0.001811	0.000003	0.818245	0.000895	0.001135	0.000001	0.687170	0.000489	0.000632	0.0000004	**0.882727**
	XGB	0.001512	0.001960	0.000004	0.800675	0.001242	0.001513	0.000002	0.452649	0.000639	0.000845	0.0000007	0.837329
Tensile strength	DT	0.016628	0.022697	0.000515	0.590361	0.006672	0.008940	0.000080	0.723862	0.006082	0.007326	0.000054	0.773773
	RF	0.012234	0.016805	0.000282	0.775445	0.008558	0.010368	0.000107	0.628581	0.005129	0.007002	0.000049	0.793313
	KNN	0.008885	0.012752	0.000163	**0.870693**	0.007962	0.010271	0.000105	0.635516	0.006544	0.010173	0.000103	0.563731
	ETR	0.010041	0.012884	0.000166	0.867995	0.006175	0.008649	0.000075	**0.742649**	0.005094	0.007331	0.000054	0.773463
	GDB	0.011410	0.015148	0.000229	0.818949	0.007563	0.009571	0.000092	0.690841	0.004166	0.005284	0.000028	**0.883173**
	XGB	0.012653	0.015314	0.000235	0.787834	0.009847	0.012563	0.000158	0.450633	0.003998	0.005258	0.000028	0.791195
Reduction area (%)	DT	0.177769	0.239443	0.057333	0.934331	0.226352	0.328100	0.107649	0.896449	0.243932	0.336854	0.113470	0.906227
	RF	0.071011	0.109865	0.012070	0.982421	0.131354	0.177339	0.031449	0.962029	0.098259	0.165782	0.027484	0.947777
	KNN	0.153361	0.195015	0.038031	0.964741	0.119214	0.155425	0.024157	0.969278	0.152350	0.211373	0.044679	0.955317
	ETR	0.047161	0.080472	0.006476	0.994922	0.071333	0.110662	0.012246	0.986186	0.088118	0.155449	0.024164	0.982710
	GDB	0.033056	0.038810	0.001506	**0.998238**	0.027657	0.038668	0.001495	**0.998921**	0.030112	0.042536	0.001809	**0.994188**
	XGB	0.040683	0.046455	0.002158	0.997839	0.039471	0.056555	0.003198	0.996559	0.033912	0.043912	0.001928	0.994120
Elongation (%)	DT	0.171304	0.249831	0.062416	0.934304	0.143370	0.222217	0.049381	0.844626	0.143963	0.217608	0.047353	0.873720
	RF	0.114111	0.142401	0.020278	0.986169	0.135815	0.170775	0.029165	0.954608	0.190780	0.238928	0.057087	0.969414
	KNN	0.138149	0.160050	0.025616	0.956421	0.120210	0.159284	0.025372	0.965133	0.143963	0.175044	0.030640	0.950278
	ETR	0.039676	0.050998	0.002601	0.992580	0.062035	0.097474	0.095013	0.982325	0.061156	0.077087	0.005942	0.973108
	GDB	0.011072	0.041344	0.001709	**0.998274**	0.000026	0.000031	0.096265	**0.997842**	0.023054	0.086185	0.007428	**0.997986**
	XGB	0.014230	0.041929	0.001758	0.997527	0.020513	0.046513	0.021635	0.995384	0.023737	0.086205	0.007431	0.997854

Significant values are in [bold].

**Table 8:** Hardness and impact of ABS, PLA and PETG.

		PLA				ABS				PETG			
		Performance metrics				Performance metrics				Performance metrics			
Hardness	Models	MAE	MSE	RMSE	R^2^	MAE	MSE	RMSE	R^2^	MAE	MSE	RMSE	R^2^
	DT	0.4158	0.5292	0.28	0.6785	0.4773	0.597	0.357	0.604	0.6844	0.923	0.85	0.642
	RF	0.192	0.2715	0.0737	0.9153	0.264	0.317	0.1005	0.936	0.5947	0.675	0.4569	0.6101
	KNN	0.233	0.3213	0.1032	0.8814	0.6488	0.8117	0.659	0.582	0.4391	0.5434	0.2953	**0.748**
	ETR	0.1394	0.193	0.0373	**0.9573**	0.2161	0.2679	0.0717	**0.955**	0.4956	0.5678	0.3225	0.7248
	GDB	0.2507	0.3582	0.1283	0.8528	0.2477	0.287	0.082	0.948	0.5595	0.6737	0.4539	0.6126
	XGB	0.3519	0.3518	0.1238	0.8579	0.2697	0.302	0.0909	0.943	0.5182	0.6405	0.4102	0.6499
Impact	DT	0.6667	1.1547	1.3334	0.9325	0.6308	0.7864	0.6184	0.975	0.32	0.505	0.256	0.981
	RF	0.6696	0.7808	0.6097	0.9692	0.5254	0.802	0.6432	0.974	0.231	0.2725	0.0742	0.995
	KNN	0.9517	1.2136	1.4728	0.9254	2.8308	3.5492	12.296	0.524	2.548	3.532	12.48	0.74
	ETR	0.6509	0.8756	0.7668	0.9612	0.4115	06,084	0.3702	0.985	0.2867	0.4787	0.2291	0.978
	GDB	0.424	0.5765	0.3324	**0.9832**	0.372	0.5829	0.3397	**0.986**	0.1248	0.221	0.0489	**0.995**
	XGB	0.8149	1.0528	1.1083	0.9438	0.716	0.9738	0.9483	0.962	0.2419	0.3729	0.139	0.9896

Significant values are in [bold].

### The results of the tensile test

The Tensile test carried on specimens of PLA, ABS, and PETG to test four properties such as Load at Peak, Tensile Strength, Reduction Area (%) and Elongation (%) of each specimen based on 11 input parameters reported in Table 2. As part of this, Tensile test experimental data of all these specimens were recorded as datasets ($d_{1}$, $d_{4}$, and $d_{7}$) and its statistics were reported in Table 7.

The results of the PLA based specimen reported in Table 7 indicated that the KNN and GDB performed better than all other models. The KNN reported low error rate and high R^2^-Score of 0.870692 and 0.870693 in predicting Load at Peak and tensile strength. Likewise, GDB outperformed all other models with low error rate and high R^2^-Score of 0.998238 and 0.998274 in predicting the percentage of reduction area (%) and elongation (%). Furthermore, the results of a specimen based on ABS material reported in Table 7 denoted that the performance of ETR and GDB models were good. The ETR performed well with low error rate and high R^2^-Score of 0.741528 and 0.742649 in predicting the properties like load at peak and tensile strength. Furthermore, GDB model performed better in predicting reduction area (%) and elongation (%) with an R^2^-Score of 0.998921 and 0.997842. Furthermore, the results of a specimen based on PETG material reported in Table 7 denoted that the performance of GDB model outperformed other models in predicting load at peak, tensile strength, reduction area (%), elongation (%) with an R^2^-Score of 0.882727, 0.883173, 0.994188, and 0.997986 respectively on PETG material.

**Fig. 10:**
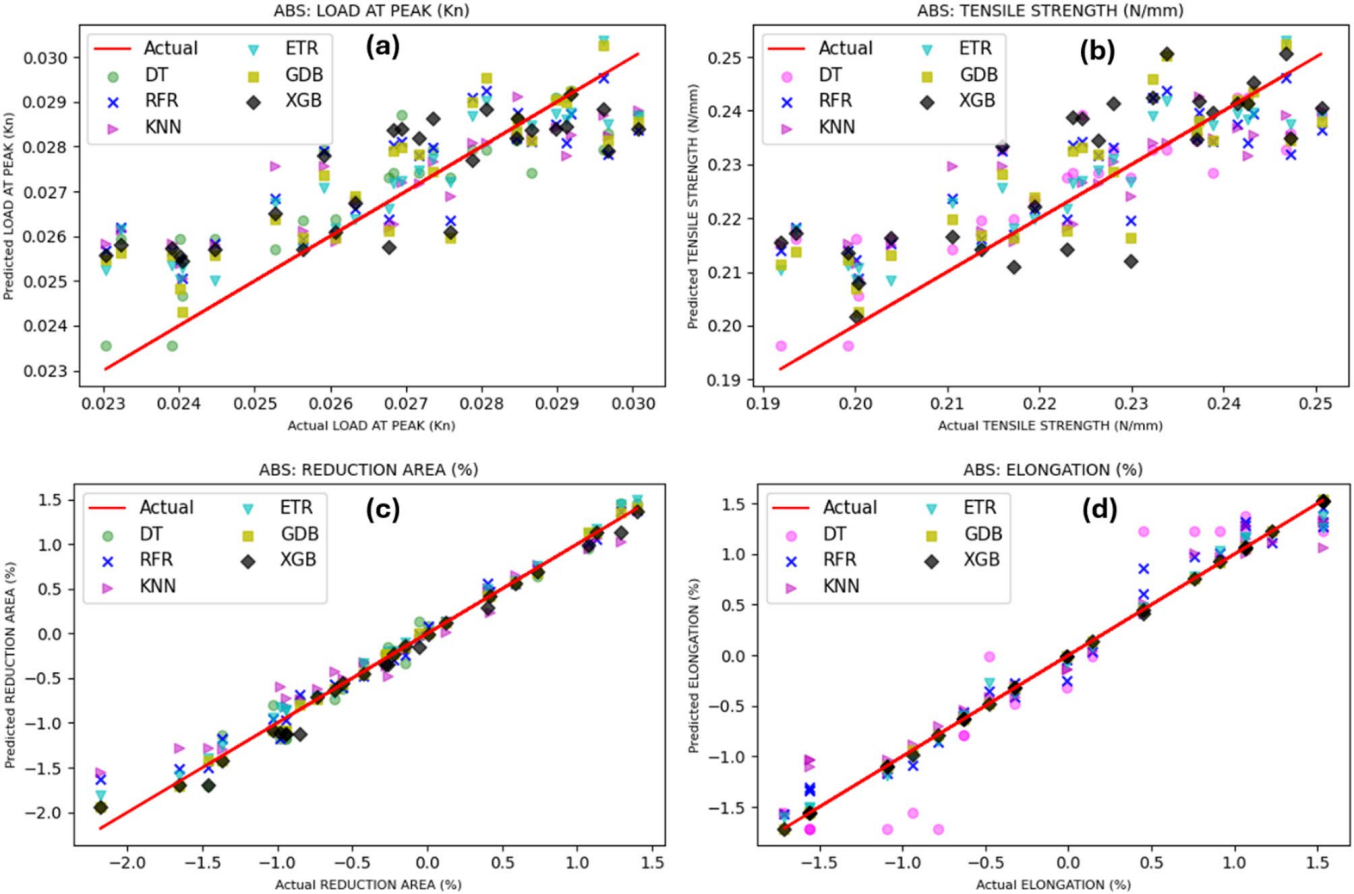
Comparison of actual vs predicted of ABS material: (**a**) Load at Peak, (**b**) Tensile Strength, (**c**) Reduction Area (%), and (**d**) Elongation (%).

**Fig. 11:**
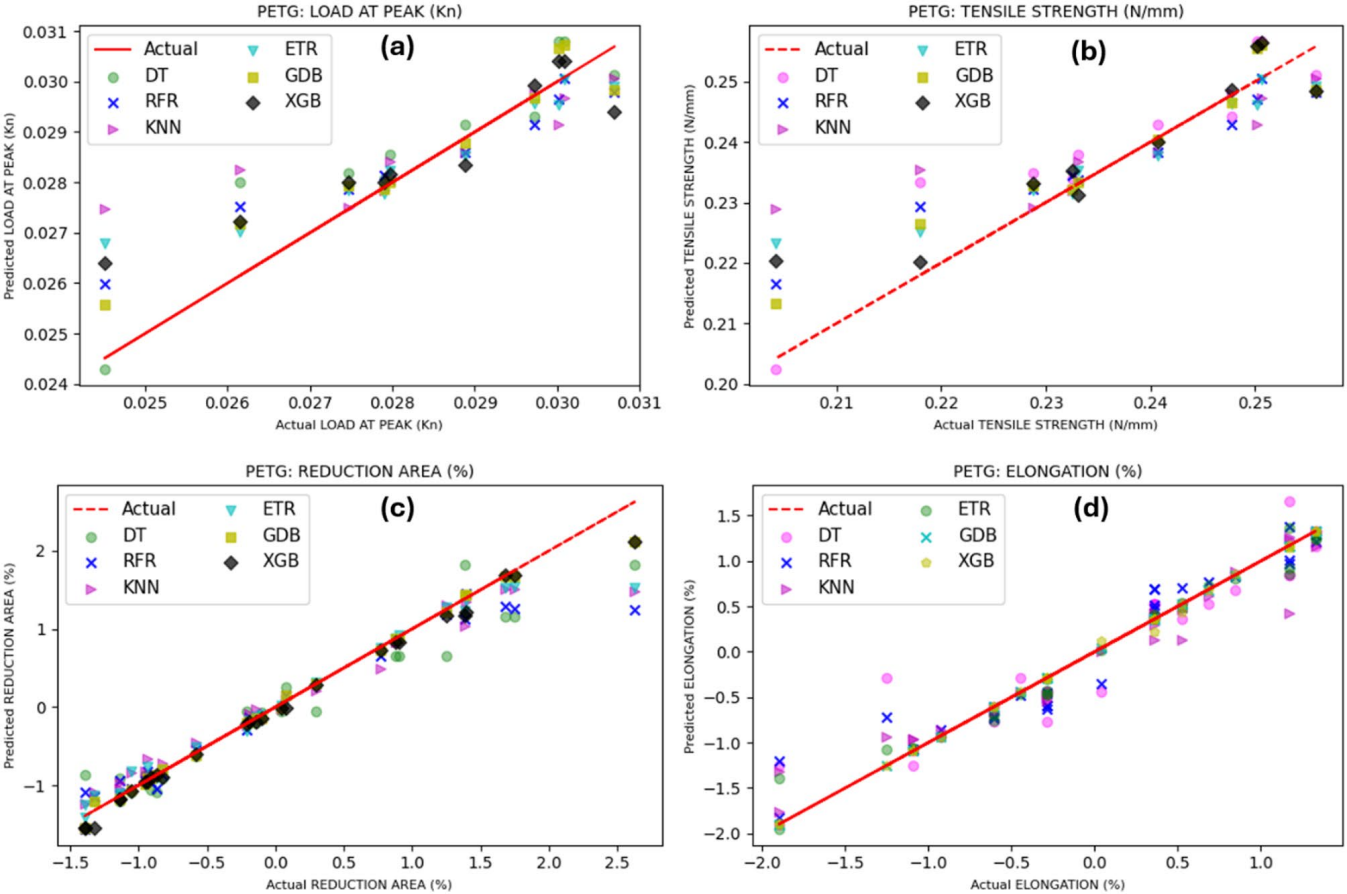
Comparison of actual vs predicted of PETG material: (**a**) Load at Peak, (**b**) Tensile Strength, (**c**) Reduction Area (%), and (**d**) Elongation (%).

Furthermore, Fig. 8 shows the actual and predicted properties of the Tensile test. Figure 8a and b shows performance comparison of load at peak and tensile strength properties of PLA specimens by all ML models. From Fig. 8a and b, it was observed that the KNN model performance is close to the actual values of load at peak and tensile strength. Moreover, from Fig. 8c and d indicated that the GDB model predicted values were close to the actual values of reduction area and elongation properties . In addition, Fig. 10 reported comparison of actual with predicted results of a specimen prepared by ABS material. Figure 10a and b reported that the ETR predicted values were closer to the actual values of load at peak and tensile strength properties. Similarly, Fig. 10c and d reported that the GBD predicted values were closer to the actual values of reduction area and elongation properties. Moreover, Fig. 11 reported comparison of actual values with predicted results of a specimen prepared by PETG material. Figure 11a, b, c and d reported that the GDB predicted values were closer to the actual values of load at peak, tensile strength, reduction area and elongation properties.

### The results of hardness test

**Fig. 12:**
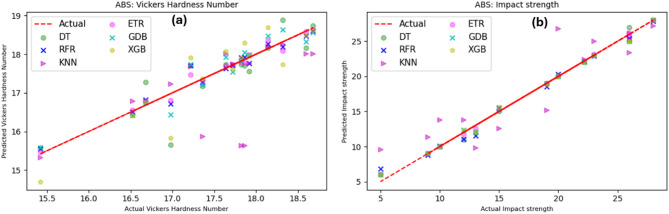
Comparison of actual vs predicted of ABS material: (**a**) Hardness, and (**b**) Impact.

**Fig. 13:**
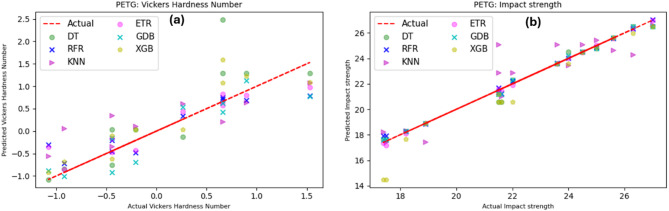
Comparison of actual vs predicted of PETG material: (**a**) Hardness, and (**b**) Impact.

The hardness tests conducted on all three specimens yielded results, which are presented in Table 8. The results show that the ETR model exhibits excellent predictive accuracy, attaining a highest R^2^ value of 0.9573 for PLA and 0.955 for ABS. Furthermore, the KNN algorithm demonstrates effective prediction of the hardness of the PETG specimen, achieving an R^2^ value of 0.748. Figures 9a, 12a, and 13a present the comparison between the experimentally measured and predicted hardness values for PLA, ABS, and PETG specimens, respectively. The data depicted in Figs. 12a and 13a indicate that the predicted values from the ETR model closely align with the actual hardness measurements. In contrast, Fig. 13a demonstrates that the KNN predictions also approximate the observed values.

### The results of impact test

The impact test results were reported in Table 8. The results indicated that the GDB outperformed all other models in predicting impact of all three specimens with high R^2^-score ranging from 0.98 to 0.99. Furthermore, Figs. 9b, 12b, and 13b reported that predicted values of the GDB model were close to the actual values of impact experimental results.

### Validation of proposed ML models

The ML models were validated through hyperparameter tuning, cross-validation, and error analysis to identify optimal parameter settings that yield high predictive accuracy with minimal error. The tree (DT, RF, and ETR), distance-based model KNN, and boosting (GDB, and XGB) based models have different training mechanics, so these models employed distinct hyperparameter grids per model family to control bias and variance. Hence, different hyperparameter tuning metrics such as learning rate (LE), maximum depth (MD), minimum number of sample leaves (SL), number of neighbours (NN), and number of estimators (NE) were employed to determine best parameters for each model. The NA indicates that the parameter is not applicable to that model, and None indicates that the model considered nothing for that parameter.

The best hyperparameters for each model are determined through best R^2^-score of the model and these are reported in Tables 9, 10 and 11 for Tensile, Hardness, and Impact of all three materials. Furthermore, the fivefold cross validation evaluated through MAE, RMSE, and R^2^-score with mean and standard deviation. The results on Tensile reported in Table 9 shows that the GDB based MTML excelled very low MAE and RMSE with high R^2^-score of 0.998 with minimal variance. Moreover, the result on Hardness as in Table 10 shows that the ETR gave the lowest errors (MAE = 0.18 and RMSE = 0.22) and highest R^2^-score = 0.93 with modest variance. The RF close behind ETE and KNN lagged higher error and variance. Furthermore, the results on Impact as reported in Table 11 shows ETR again best RMSE of 0.48 and R^2^-score of 0.99 with low variance.

**Table 9:** Hyperparameter tuning and fivefold cross validation on Tensile.

Models	Best parameters					Model evolution through fivefold cross validation		
	LE	MD	NE	SL	NN	MAE mean ± SD	RMSE mean ± SD	R^2^-score mean ± SD
DT	NA	5	NA	1	NA	0.508 ± 0.154	0.719 ± 0.191	0.796 ± 0.115
KNN	NA	NA	NA	NA	5	0.559 ± 0.071	0.728 ± 0.111	0.621 ± 0.131
RF	NA	10	200	1	NA	0.316 ± 0.059	0.445 ± 0.115	0.878 ± 0.077
ETR	NA	None	200	1	NA	0.254 ± 0.051	0.363 ± 0.072	0.922 ± 0.028
GDB	0.1	NA	300	NA	NA	**0.062 ± 0.017**	**0.132 ± 0.053**	**0.998 ± 0.001**
XGB	1.0	NA	200	NA	NA	0.097 ± 0.011	0.201 ± 0.037	0.996 ± 0.001

Significant values are in [bold].

**Table 10:** Hyperparameter tuning and fivefold cross validation on hardness.

Models	Best parameters					Model evolution through fivefold cross validation		
	LE	MD	NE	SL	NN	MAE mean ± SD	RMSE mean ± SD	R^2^-score mean ± SD
DT	NA	5	NA	1	NA	0.333 ± 0.097	0.430 ± 0.124	0.744 ± 0.133
KNN	NA	NA	NA	NA	3	0.543 ± 0.110	0.704 ± 0.156	0.428 ± 0.167
RF	NA	10	400	1	NA	0.201 ± 0.062	0.254 ± 0.067	0.916 ± 0.056
ETR	NA	None	400	1	NA	**0.180 ± 0.045**	**0.222 ± 0.048**	**0.932 ± 0.045**
GDB	0.03	NA	300	NA	NA	0.198 ± 0.034	0.254 ± 0.040	0.914 ± 0.043
XGB	0.1	NA	400	NA	NA	0.286 ± 0.073	0.357 ± 0.110	0.816 ± 0.122

Significant values are in [bold].

**Table 11:** Hyperparameter tuning and fivefold cross validation on impact.

Models	Best parameters					Model evolution through fivefold cross validation		
	LE	MD	NE	SL	NN	MAE mean ± SD	RMSE mean ± SD	R^2^-score mean ± SD
DT	NA	None	NA	1	NA	0.669 ± 0.391	0.938 ± 0.436	0.973 ± 0.035
KNN	NA	NA	NA	NA	3	3.233 ± 0.634	3.758 ± 0.634	0.709 ± 0.107
RF	NA	None	400	1	NA	0.576 ± 0.132	0.782 ± 0.157	0.986 ± 0.009
ETR	NA	10	200	1	NA	**0.354 ± 0.125**	**0.475 ± 0.170**	**0.994 ± 0.004**
GDB	0.03	NA	300	NA	NA	0.486 ± 0.191	0.721 ± 0.240	0.986 ± 0.012
XGB	1.0	NA	400	NA	NA	0.585 ± 0.301	0.852 ± 0.418	0.977 ± 0.030

Significant values are in [bold].

## Conclusion

This study demonstrated the effectiveness of the MTML framework for predicting various mechanical properties of FDM-fabricated polymer components using datasets generated experimentally for PLA, ABS, and PETG materials. By jointly modeling the correlated mechanical responses from tensile, hardness, and impact tests, this approach effectively captures complex, non-linear relationships that conventional single-output prediction strategies fail to represent adequately.

Despite the notable predictive performance demonstrated by various ensemble regression models, this study is not without its limitations. The experimental datasets, particularly those pertaining to hardness and impact testing, are relatively small, which may restrict the generalizability of the developed models beyond the specific material systems and parameter ranges that were examined. Furthermore, the modeling framework is based on experimentally observed variables rather than real-time process parameters. This positions the current study towards estimating post-fabrication performance, rather than facilitating closed-loop process control.

Future research endeavours should focus on addressing the identified limitations by incorporating larger and more diverse datasets, expanding the framework to encompass additional materials and processing conditions, and integrating systematic cross-validation alongside hyperparameter optimization strategies. Moreover, the integration of MTMLM with in-situ sensing data has the potential to facilitate real-time predictions and adaptive control. This integration would support the advancement of digital twin-enabled additive manufacturing systems.

From an industrial perspective, the proposed MTML framework provides significant practical value as a decision-support tool for manufacturing based on FDM. This framework facilitates offline parameter selection, assessment of quality consistency, and performance estimation, thereby minimizing the necessity for trial-and-error experimentation and reducing material waste. The capacity to simultaneously predict multiple mechanical properties significantly enhances both reliability and repeatability. This approach is particularly pertinent to the production of functional components as well as to data-driven manufacturing processes that adhere to the principles of Industry 4.0.

## Data Availability

The datasets generated during and/or analyzed during the current study are available from the corresponding author on reasonable request.
